# Disparities by race, age, and sex in the improvement of survival for lymphoma: Findings from a population-based study

**DOI:** 10.1371/journal.pone.0199745

**Published:** 2018-07-11

**Authors:** Fahad Mukhtar, Paolo Boffetta, Bashir Dabo, Jong Y. Park, Chi T. D. Tran, Thuan V. Tran, Huong Thi-Thanh Tran, Madison Whitney, Harvey A. Risch, Linh C. Le, Wei Zheng, Xiao-Ou Shu, Hung N. Luu

**Affiliations:** 1 Department of Epidemiology and Biostatistics, College of Public Health, University of South Florida, Tampa, FL, United States of America; 2 Tisch Cancer Institute, Icahn School of Medicine, Mount Sinai School of Medicine, New York, NY, United States of America; 3 Department of Cancer Epidemiology, H. Lee Moffitt Cancer Center and Research Institute, Tampa, FL, United States of America; 4 Vietnam Colorectal Cancer and Research Program, Vinmec Healthcare System, Hanoi, Vietnam; 5 Vietnam National Cancer Hospital, Hanoi, Vietnam; 6 Vietnam National Institute for Cancer Control, Hanoi, Vietnam; 7 Department of Chronic Disease Epidemiology, Yale School of Public Health, Yale University, New Haven, CT, United States of America; 8 Vinmec University of Health Sciences Project, Vinmec Healthcare System, Hanoi, Vietnam; 9 Division of Epidemiology, Department of Medicine, Vanderbilt Epidemiology Center, Vanderbilt-Ingram Cancer Center, Vanderbilt University School of Medicine, Nashville, TN, United States of America; 10 Department of Epidemiology, University of Pittsburgh Graduate School of Public Health, Pittsburgh, PA, United of States America; 11 Currently at the Division of Cancer Control and Population Sciences, University of Pittsburgh Cancer Institute, Pittsburgh, PA, United of States America; Universita degli Studi di Roma La Sapienza, ITALY

## Abstract

**Objective:**

To evaluate improvement in survival of lymphoma patients from 1990 to 2014, stratified by age, sex and race using Surveillance Epidemiology and End-Result Survey Program (SEER) data.

**Study design and setting:**

We identified 113,788 incident lymphoma cases from nine SEER cancer registries were followed up for cause-specific mortality from lymphoma. Cox proportional hazard regression was used to estimate hazard ratios (HRs) and their respective 95% confidence interval (CIs) for various time periods within groups stratified by race, age and sex.

**Results:**

Five-year survival for Hodgkin’s lymphoma (HL) was 89% for patients 20–49 years of age. For this age group, compared to 1990–1994, survival significantly improved in 2000–2004 (HR = 0.65; 95% CI: 0.54–0.78), 2005–2009 (HR = 0.46, 95% CI: 0.38–0.57) and 2010–2014 (HR = 0.29, 95% CI: 0.20–0.41). Hodgkin’s lymphoma patients aged 75–85 years had 5-year survival of 37% and in these patients, compared to 1990-1994, survival only improved from 2005 onward (HR = 0.67, 95% CI: 0.50–0.90). In patients with non-Hodgkin’s Lymphoma (NHL), all age groups showed survival improvements between 1990–1994 period and 2010–2014 period. Improvements in HL and NHL survival were seen for all race categories and both genders.

**Conclusion:**

Survival among US lymphoma patients has improved substantially between 1990–1994 period and 2010–2014 period, though disease-specific mortality was still higher in older age groups.

## Introduction

Survival of patients with lymphoma has substantially improved over the last few decades due to advances in therapy, particularly approaches that target pathways of malignancy and lead to partial or complete recovery [1,2]. Non-Hodgkin’s Lymphoma (NHL), the more prevalent form of the two major types of lymphoma, has a 5-year survival of 70%, while Hodgkin’s lymphoma (HL) has a 5-year survival of 86% [3]. Advances in HIV/AIDS treatment has also led to reduced incidence of HL and NHL [4,5].

Prevailing incidence rates and survival of lymphoma vary by race, sex and age. For example, NHL is more common in adults aged 65–74, and patients aged 75 to 84 have the highest mortality [3]. Some studies have found no significant differences in survival between men and women [6,7] while others suggest that females have better survival than males [8–10]. Regarding to race, white males and white females have the highest age-adjusted incidence rates and mortality [3].

Also, differences in access to advanced therapy and in participation in clinical trials for new therapies have been found in several studies [11–14]. Specifically, blacks, females and elderly patients are less likely to participate in clinical trials and less likely to have access to better treatment modalities [14,15]. For example, Shavers et al. [12] found that racial disparity still persisted with respect to access to quality care, cancer diagnostic services and service delivery. Another study by Tao et al. [11] also showed that low socio-economic status and lack of adequate health insurance coverage were associated with poorer survival of lymphoma patients. These studies suggest that even though survival has generally improved over time, improvement may not necessarily be the same for all patients. While substantial efforts have been made to determine survival and its predictors of lymphoma, there is a limited understanding the secular trends in survival of lymphoma patients. To address this gap, we examined lymphoma patient survival by age, sex and race across 25-year span using SEER data during 1990–2014 period and explored possible reasons for such differences.

## Materials and methods

We obtained data from the National Cancer Institute’s cancer registry program, the Surveillance Epidemiologic and End-Result Survey (SEER) [3]. Accordingly, the SEER data are collected from 18 cancer registries and represent 28% of the US population. SEER data contain cancer incidence and mortality information, as well as individual patient demographics and clinical characteristics including cancer staging, grade and treatment. Data from nine cancer registries in the SEER program was used for the current analysis, including Atlanta, Georgia; Connecticut; Detroit, Michigan; Hawaii; Iowa; New Mexico; San Francisco-Oakland, California; Seattle-Puget Sound, Washington; and Utah.

We identified patients with first primary diagnoses of lymphoma from 1990 to 2014 (97,300 cases of NHL and 16,488 cases of HL) comprising of the following ICD-0-3 codes: 9590–91 9596–97, 9650–55, 9659, 9661–67, 9670–71, 9673, 9675, 9678–80, 9684, 9687–91, 9695, 9698–9705, 9708–09, 9712, 9714, 9716–19, 9726–29, 9735, 9737–38, 9811–12, 9816, 9818, 9823, 9827 and 9837. Patients less than 20 years of age or older than 85 years, patients with unknown age and those whose diagnosis was only based on autopsy report or based on death certificate were excluded. Other information that was obtained included age at diagnosis, sex, marital status, race and ethnicity, grade staging based on Ann Arbor classification, and SEER registry location. The SEER race data comprised 5 categories: White, African American, American Indian/Alaskan Native, Asian/Pacific Islander, and unknown. Because the number of American Indian/Alaskan Native patients was small, they were combined with “unknown” into a single group as “other”. Age at diagnosis was categorized into: 20–49, 50–64, 65–74 and 75–84 years. Marital status was recoded such that small sized categories including divorced, widowed, domestic partner and separated were grouped as “other”. Ann Arbor classification for staging included categories: 1) stage I disease involving a single lymph node; 2) stage II disease involving two or more lymph nodes on the same side of the diaphragm; 3) stage III disease involving lymph nodes on both sides of the diaphragm; and 4) stage IV disseminated disease.

The primary outcome was SEER-identified disease-specific mortality from lymphoma. Survival time was defined from date of diagnosis to date last known to be alive, date of death or at December 31, 2014. Patients still alive on December 31, 2014, or who had died of other causes were censored at date of death. For the baseline period 1990 to 1994, cancer-specific survival was estimated using Kaplan-Meier (product-limit) survival tables at 1, 3, and 5 years according to categories of age, sex, and race. We used Cox proportional hazards regression to calculate Hazard Ratios (HRs) and their 95% confidence intervals (CIs) for cancer specific mortality for patients diagnosed during 1995 to 1999, 2000 to 2004, 2005 to 2009, and 2010 to 2014 periods and compared with those diagnosed at the baseline (1990 to 1994 period) for each sex, race and age group. Interaction between year of diagnosis with age, sex and race was evaluated using likelihood ratio test [16,17]. We also calculated HRs and 95% CIs for each 5-year increment from 1990 to 2014 within each stage of cancer and age group of patients (i.e., 3-way interaction between year of diagnosis, stage and age group). Variables included in the final models were age, race, sex, marital status, stage and SEER registry site. The proportional hazard assumption was evaluated using Schoenfeld residual plots and log-log survival plots for each variable that were evaluated in the current analysis. Because individuals diagnosed in the later years of follow up with lymphoma may not have had enough follow up time to die as a result of their disease, we performed a sensitivity analysis by excluding cases diagnosed from 2010 to 2014 and repeating the survival analysis. We compared the results of both analyses to see if there were differences in the overall conclusion. All statistical analyses were performed using SAS 9.4 (SAS Institute Inc., Cary, NC) and SEER*stat 8.3.2. All reported *P*-values were two-sided and the threshold 0.05 was considered a significant level.

## Results

We identified a total of 133,788 eligible lymphoma patients, diagnosed from 1990 to 2014. During the period of 1990–1994, survival rates were lowest for African—American patients in both HL (5-year survival: 73%) and NHL (40%). Men had poorer survival for lymphoma in general (5-year survival: 48%), HL (80%) and NHL (42%). Survival was lowest for the oldest age group (75–85) while those 50–64 years had the best overall survival for NHL and those aged 20–49 years had the best survival for HL (Table 1).

**Table 1:** Cancer-specific survival (Percentage) for lymphoma patients by age group, race/ethnicity, and sex diagnosed in 9 SEER registries (1990–1994).

Survival time	Age				Race				Sex	
	20–49	50–64	65–74	75–85	White	African American	Asian	Other	Men	Women
**Lymphoma**										
1 year	74.30	78.28	72.61	63.79	73.75	66.10	69.48	82.94	68.99	78.08
3 years	64.74	63.77	57.03	46.27	60.54	51.41	56.11	71.91	54.78	65.83
5 years	61.62	56.21	48.34	37.93	54.19	46.56	50.26	70.02	48.67	59.53
**Hodgkin Lymphoma**										
1 year	97.00	89.47	74.61	67.57	93.28	89.03	85.58	100	92.49	92.97
3 years	91.34	80.14	60.40	44.35	85.99	79.19	81.94	92.31	83.94	86.87
5 years	88.86	72.52	52.70	36.72	82.65	73.25	78.22	92.31	80.02	83.69
**Non-Hodgkin Lymphoma**										
1 year	64.12	77.41	72.51	63.61	70.24	60.83	68.13	80.64	64.47	73.00
3 years	52.80	62.52	56.86	46.36	55.92	44.98	53.99	69.24	49.21	59.26
5 years	49.37	54.97	48.12	37.99	48.97	40.36	47.95	67.11	42.73	52.57

**Fig 1:**
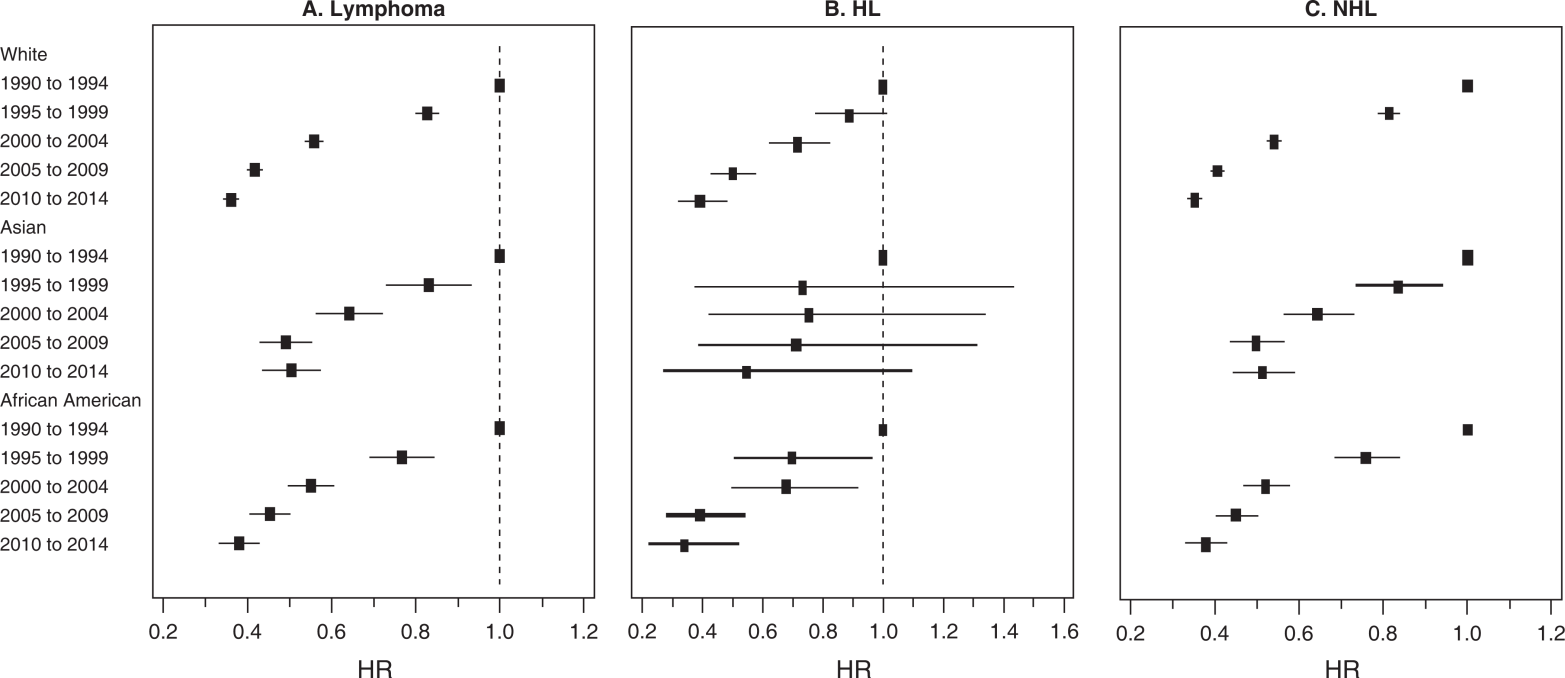
Forest plots showing Multivariate-Adjusted Hazard Ratios (HRs) and 95%CIs for cancer-specific death (due to lymphoma) by race and year of Diagnosis in 9 SEER Registries (1990–2014). HL: Hodgkin’s Lymphoma; NHL: Non-Hodgkin’s Lymphoma; HR: Hazard ratio.

Survival improvement differed by race over time from 1995 to 2014 (Table 2 and Fig 1). Whites had significant improvement in survival for both HL and NHL (Table 2 and Fig 1B–1C). For HL, there has been no improvement in survival for Asian Americans from 1995 to 2014. In African Americans with HL, survival increased significantly during 1995–1999 period, compared to 1990–1994 period. However, compared to 1995–1999 period, there has been no significant difference in survival in all the time periods from 2000 to 2014 (Fig 1B). For NHL, survival improved for both whites and African Americans. In Asian American, survival improved over the entire study period. However, survival from 2010 to 2014 was not significantly different from that in 2005–2009 period (Fig 1C).

**Table 2:** Multivariate-Adjusted Hazard Ratios (HRs) and 95% CIs for cancer-specific death (due to lymphoma) by race and year of diagnosis in 9 SEER Registries (1990–2014).

Race	Lymphoma	Hodgkin Lymphoma	Non-Hodgkin Lymphoma
	HR (95% CI)	HR (95% CI)	HR (95% CI)
**White**			
1990–1994	Ref	Ref	Ref
1995–1999	0.82 (0.80–0.85)	0.89 (0.78–1.01)	0.82 (0.79–0.84)
2000–2004	0.56 (0.54–0.58)	0.71 (0.62–0.82)	0.54 (0.52–0.56)
2005–2009	0.42 (0.40–0.43)	0.50 (0.43–0.58)	0.41 (0.39–0.43)
2010–2014	0.36 (0.34–0.38)	0.39 (0.32–0.48)	0.35 (0.34–0.37)
**Asian**			
1990–1994	Ref	Ref	Ref
1995–1999	0.83 (0.73–0.94)	0.73 (0.37–1.43)	0.84 (0.74–0.95)
2000–2004	0.64 (0.56–0.72)	0.75 (0.42–1.34)	0.64 (0.57–0.73)
2005–2009	0.49 (0.43–0.56)	0.71 (0.38–1.31)	0.50 (0.44–0.57)
2010–2014	0.50 (0.44–0.58)	0.54 (0.27–1.10)	0.51 (0.45–0.59)
**African American**			
1990–1994	Ref	Ref	Ref
1995–1999	0.77 (0.71–0.85)	0.70 (0.50–0.96)	0.76 (0.68–0.84)
2000–2004	0.55 (0.50–0.61)	0.68 (0.50–0.92)	0.52 (0.47–0.58)
2005–2009	0.45 (0.41–0.50)	0.39 (0.28–0.55)	0.45 (0.40–0.52)
2010–2014	0.38 (0.34–0.43)	0.34 (0.22–0.52)	0.38 (0.33–0.43)

CI: confidence interval; HR: Hazard ratio

**Fig 2:**
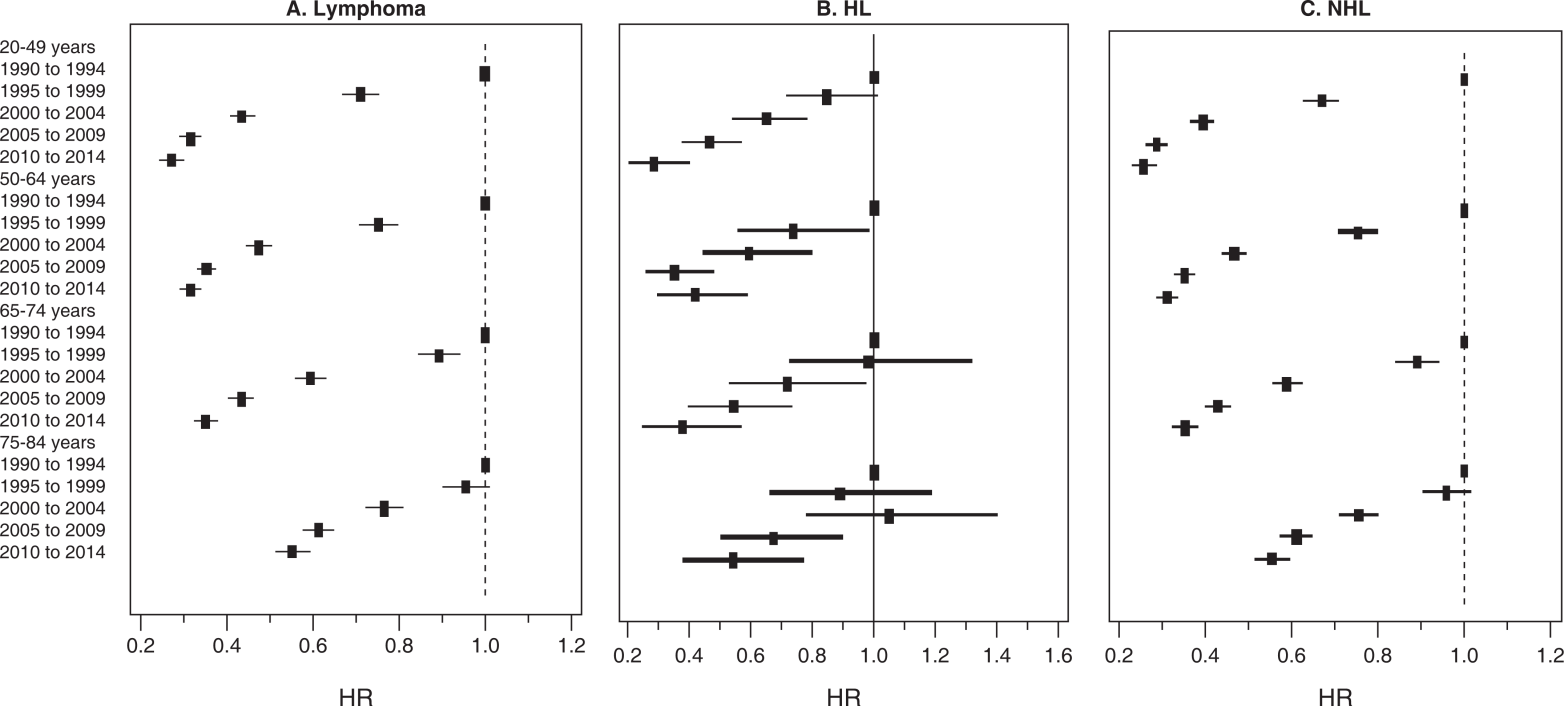
Forest plots showing Multivariate-Adjusted Hazard Ratios (HRs) and 95%CIs for Cancer-Specific Death (due to lymphoma), by Age Group and Year of Diagnosis in 9 SEER Registries (1990–2014). HL: Hodgkin’s Lymphoma; NHL: Non-Hodgkin’s Lymphoma; HR: Hazard ratio.

Table 3 evaluated the cancer-specific death by age group and year of diagnosis. We observed an improvement in survival across age groups for all patients with lymphoma. Those with younger age had higher improvement in survival (Table 3 and Fig 2A). For example, compared to 1990–1994 period, the hazard ratio in 2010–2014 period for those aged 20–49 was 0.27 (95% CI: 0.24–0.30) while it was 0.55 (95% CI: 0.51–0.59) for those 75–85 years old. For HL, survival began to significantly improve for the younger age groups much earlier compared to the older age groups (Table 3 and Fig 2B). For those aged 20–49, 50–64, and 65–74, survival during 2000–2004 period was significantly better compared to survival among these age group during 1990–1994 period (HR = 0.65, 95% CI: 0.54–0.78; 0.60, (0.45–0.80) and 0.72, (0.53–0.97); respectively). In those who were older than 75 years of age, survival appeared to significantly improve only during 2005–2009 period (HR = 0.67, 95% CI: 0.50–0.90; reference group: 1990–1994 period). For NHL, survival began to significantly improve during 1995–1999 period for all ages except those older than 75 years where survival started to improve in 2000–2004 period (HR = 0.75, 95% CI: 0.71–0.80) (Table 3 and Fig 2C).

**Table 3:** Multivariate-Adjusted Hazard Ratios (HRs) and 95%CIs for cancer-specific death (due to lymphoma), by age group and year of diagnosis in Nine SEER Registries During 1990–2014 Period.

Age Group and Period	Lymphoma	Hodgkin Lymphoma	Non-Hodgkin Lymphoma
	HR (95% CI)	HR (95% CI)	HR (95% CI)
**20–49**			
1990–1994	Ref.	Ref.	Ref.
1995–1999	0.71 (0.67–0.75)	0.85 (0.71–1.01)	0.67 (0.63–0.71)
2000–2004	0.43 (0.41–0.47)	0.65 (0.54–0.78)	0.39 (0.36–0.42)
2005–2009	0.32 (0.29–0.34)	0.46 (0.38–0.57)	0.29 (0.26–0.31)
2010–2014	0.27 (0.24–0.30)	0.29 (0.20–0.40)	0.26 (0.23–0.29)
**50–64**			
1990–1994	Ref.	Ref.	Ref.
1995–1999	0.75 (0.71–0.80)	0.74 (0.55–0.98)	0.75 (0.71–0.80)
2000–2004	0.47 (0.44–0.50)	0.60 (0.45–0.80)	0.47 (0.44–0.50)
2005–2009	0.35 (0.33–0.38)	0.35 (0.26–0.48)	0.35 (0.33 0.38)
2010–2014	0.32 (0.29–0.34)	0.42 (0.29–0.59)	0.31 (0.29 0.34)
**65–74**			
1990–1994	Ref.	Ref.	Ref.
1995–1999	0.89 (0.84–0.95)	0.98 (0.72–1.32)	0.89 (0.84–0.94)
2000–2004	0.59 (0.56–0.63)	0.72 (0.53–0.97)	0.59 (0.55–0.63)
2005–2009	0.43 (0.40–0.46)	0.54 (0.40–0.74)	0.43 (0.40–0.46)
2010–2014	0.35 (0.32–0.38)	0.37 (0.25–0.57)	0.35 (0.32–0.38)
**75–84**			
1990–1994	Ref.	Ref.	Ref.
1995–1999	0.95 (0.90–1.01)	0.89 (0.66–1.19)	0.96 (0.90–1.02)
2000–2004	0.76 (0.72–0.81)	1.05 (0.78–1.40)	0.75 (0.71–0.80)
2005–2009	0.61 (0.58–0.65)	0.67 (0.50–0.90)	0.61 (0.57–0.65)
2010–2014	0.55 (0.51–0.59)	0.54 (0.38–0.77)	0.55 (0.51–0.60)

CI: confidence interval; HR: Hazard ratio

**Fig 3:**
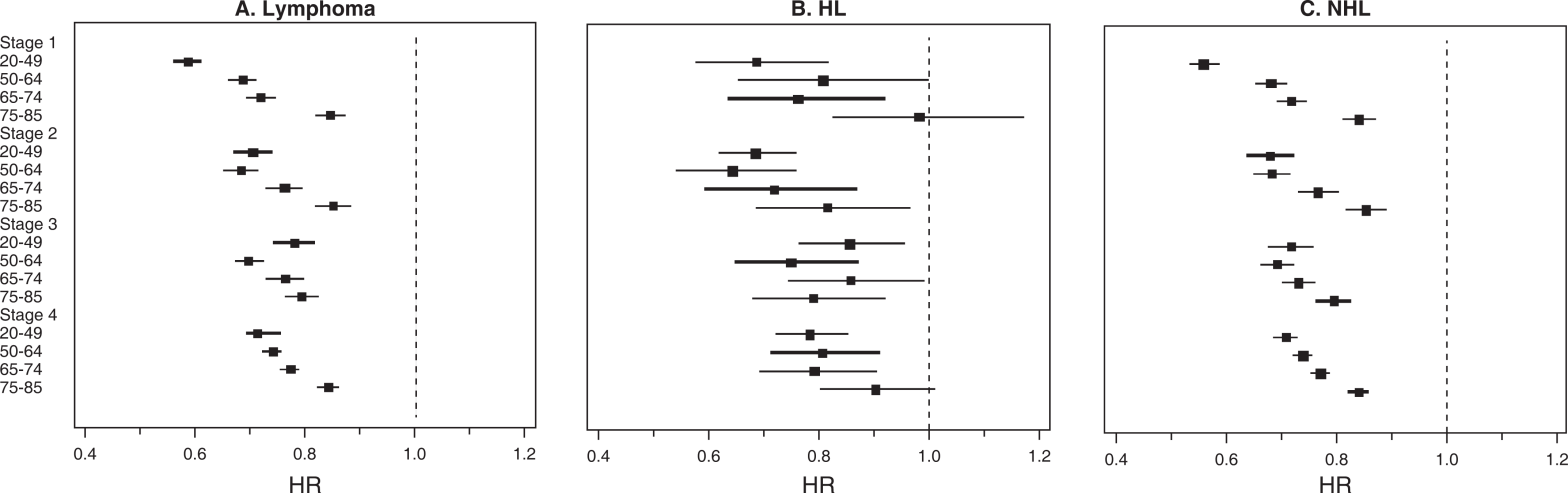
Forest plots showing Multivariate-Adjusted Hazard Ratios (HRs) and 95% CIs for cancer-specific death (due to lymphoma) by stage and age group in 9 SEER Registries (1990–2014). HL: Hodgkin’s Lymphoma; NHL: Non-Hodgkin’s Lymphoma; HR: Hazard ratio.

Table 4 shown the 3-way interaction between stage, age and year of diagnosis with survival of lymphoma, HL and NHL patients. For HL, the improvement in survival across all the stages were more pronounced in the younger age groups compared to the older age groups. However, patients at 65–74 year of age with stage IV HL disease had the most favorable survival (HR = 0.79, 95% CI: 0.69–0.91) (Table 4 and Fig 3B). There was, however, no significant statistical difference in survival for all age groups within each stage (Fig 3B). For NHL, younger aged individuals had the most favorable survival in all four stages of the disease (Fig 3C). Survival was decreased with advancing stage and within each stage, with advancing age (Fig 3C). Sensitivity analysis that excluded cases diagnosed from 2010 to 2014 showed no material difference in survival trend for the different stages of the disease (S1 Table). Indeed, younger age patients in each stage had the most favorable survival except for stage IV where adults aged 65 to 74 had the most favorable survival (HR = 0.76, 95% CI: 0.64–0.90). For NHL, improvement in survival over time occurred in all age groups for all stages but much greater in the younger patients compared to the older patients.

**Table 4:** Multivariate-Adjusted Hazard Ratios (HRs) and 95% CIs for cancer-specific death (due to lymphoma) by stage and age group in Nine SEER Registries during 1990–2014 period.

Age Group and Period	Lymphoma	Hodgkin Lymphoma	Non-Hodgkin Lymphoma
	HR (95% CI)	HR (95% CI)	HR (95% CI)
**Stage 1**			
20–49	0.59 (0.56–0.61)	0.69 (0.57–0.82)	0.56 (0.53–0.59)
50–64	0.69 (0.66–0.71)	0.81 (0.65–1.00)	0.68 (0.65–0.71)
65–74	0.72 (0.69–0.75)	0.76 (0.63–0.92)	0.72 (0.69–0.75)
75–85	0.85 (0.82–0.88)	0.98 (0.82–1.17)	0.84 (0.81–0.87)
**Stage 2**			
20–49	0.70 (0.67–0.74)	0.69 (0.62–0.76)	0.68 (0.64–0.72)
50–64	0.68 (0.65–0.72)	0.64 (0.54–0.76)	0.68 (0.65–0.72)
65–74	0.76 (0.73–0.80)	0.72 (0.59–0.87)	0.77 (0.73–0.80)
75–85	0.85 (0.82–0.89)	0.82 (0.69–0.97)	0.85 (0.82–0.89)
**Stage 3**			
20–49	0.78 (0.74–0.82)	0.86 (0.77–0.96)	0.72 (0.68–0.76)
50–64	0.70 (0.67–0.73)	0.75 (0.65–0.87)	0.69 (0.66–0.72)
65–74	0.76 (0.73–0.80)	0.86 (0.74–0.99)	0.73 (0.70–0.77)
75–85	0.79 (0.77–0.82)	0.79 (0.68–0.92)	0.80 (0.76–0.83)
20–49			
**Stage 4**			
20–49	0.71 ((0.69–0.73)	0.78 (0.72–0.85)	0.71 (0.69–0.73)
50–64	0.74 (0.72–0.76)	0.81 (0.71–0.91)	0.74 (0.72–0.76)
65–74	0.77 (0.75–0.79)	0.79 (0.69–0.91)	0.77 (0.75–0.79)
75–85	0.84 (0.82–0.86)	0.90 (0.80–1.01)	0.84 (0.82–0.86)

CI: confidence interval; HR: Hazard ratio

## Discussion

Using data from SEER, we found slower improvement in survival among older patients compared to younger patients for all lymphomas combined and for HL and NHL separately during the past 20 years in the United States. The age-related disparity in survival was most striking for NHL, for which the bigger advances in treatment were made within the study period. Notably, improved survival was highest within the period 1995 to 2004 when immunotherapy (Rituximab) was introduced to treat certain types of NHL and was commonly used later [18]. This period also corresponded to the widespread use of combination antiretroviral therapy to treat HIV/AIDS [19], which was associated with risk of poor prognosis for NHL [20].

While there is no comparable study to ours, this pattern is similar to findings from a recent study by Zeng et al. [21] in which they found that age-related gap in survival to be wider for prostate, colorectal and breast cancers, owing to the highest treatment advances among the cancers examined. Moreover, analysis based on lymphoma stage was suggestive of better improved survival among younger patients for more localized than for advanced NHL and, to a lesser extent, HL. Findings from clinical trials and other studies also showed improved NHL survival for patients with more localized disease associated with more advanced surgical techniques and adjuvant chemotherapy [22,23]. These results suggested that the age-related disparity in lymphoma survival may due to different utilization of more recent cancer treatments among the elderly.

From available evidence, older age is associated with higher rate of complications from surgery and toxicity from chemotherapy and radiotherapy [24–26]. In addition, there is preponderance of other factors that could affect the outcome of cancer treatment among the elderly, such as impaired organ function, comorbidity and malnutrition [27–29]. These may have led physicians to use less aggressive or shortened treatment courses in order to minimize harm to the elderly patients [30]. Additionally, limited participation of elderly cancer patients in clinical trials [15] has resulted in general dearth of information on how they will react to combination chemotherapy or (newer) targeted cancer treatments [31,32]. A classic dogma is that treatments that work well for younger cancer patients may be less useful or more harmful for older ones [33]. However, evidence exist to suggest otherwise, at least for some types of lymphoma [34]. Therefore, in showing that differences in lymphoma survival improvement exist based on age, our findings highlight the relevance of studies both observational and experimental, involving the elderly, to develop the most proper cancer treatments and to understand the side effect profile among elderly population of patients.

We found differences in survival improvement based on race, with Asians having the lowest rise in survival during the whole study period, especially for HL in which none of the 5-year intervals had a statistically significant increase in survival among Asians. Previous studies also reported similar racial pattern of survival for lymphomas, with whites and African Americans having more favorable survival than Asians [35–37]. However, another study on racial disparity in HL survival found Asians to have better survival compared to African Americans [38]. Remarkably, we found African Americans to have better HL survival advancement compared to whites. This is consistent with results from previous studies which showed reduced survival gap between whites and African Americans from 2000 to 2009 for follicular lymphoma [37].

Although previous studies have found females to have better survival for all non-gender dependent malignancies [39] and some types of lymphomas [40], we did not find any difference in survival improvement across the years between males and females in our analysis.

The current analysis has some limitations. First, because we only focused on analysis on those who were 20 years or older, we could not generalize our finding to young patients (i.e., less than 20 years of age). Ability to apply our results to the general US population could also be limited by the SEER program’s over sampling of foreign born and urban dwelling individuals. However, our data closely resembled the general US population in terms of key sociodemographic characteristics within the age range of participants we considered in our study. Second, we could not obtain information from the SEER registries to assess whether cancer screening and diagnosis had any influence on the results from our analysis. Lead-time bias could have occurred if screening trends were different over time between the groups we compared. However, we could not find any evidence to suggest screening trend differed over the period. Also, the fact that we observed disparity in improvement in survival due to age across almost all stages of lymphoma means the disparities could not have been only due to changes in screening practices within the study period. Other limitations include lack of information in the SEER database on many factors with potential to affect lymphoma survival. This unmeasured confounding could occur, for example, due to HIV/AIDS and other comorbidities, life style factors, socioeconomic status, health insurance coverage of the individual, among other factors. Other limitations include those that are known to occur in studies involving retrospective analysis of databases such as the SEER. Because of the possibility that some cases diagnosed in the interval 2010 to 2014 may not have had up to five-year follow up, we carried out sensitivity analysis by excluding this time interval from the analysis. However, results from these sensitivity analyses shown no material changes from current analysis.

Despite the limitations, our study has strength in many areas. To our knowledge, this is the first effort to evaluate the secular trend in survival of lymphoma patients by age, sex, and race/ethnicity groups with a largest sample size to date. Also, barring the few limitations noted earlier, the sample used is representative of the US population and findings can be generalizable. Moreover, stringent measures were taken by the SEER to ensure production of data of high quality that is reliable.

The disparities we have observed in survival improvement among lymphoma patients based on age and race may be indicating divergent levels of cancer care and/or response to treatment across categories of these variables. Therefore, while the better acknowledged causes of racial disparity are being addressed to eliminate disparity in lymphoma survival, others need to also be considered. For example, inclusion of older cancer patients and those from minority racial groups in clinical trials should be increased so that enough evidence could be obtained on them regarding new treatments. This may encourage clinicians to consider such treatments in these patients to ensure they are at par with others in access to all forms of treatment. Future research should focus on identifying other factors that could be responsible for disparity in lymphoma survival.

## Supporting information

S1 Table Multivariate-Adjusted Hazard Ratios (HRs) and 95% CIs for cancer-specific death (due to Lymphoma) by stage and age group in 9 SEER Registries (1990–2009). (DOCX)

S2 Table Baseline Demographic and Tumor Characteristics of Lymphoma Cancer Patients by Diagnosis Year in Nine SEER Registries, 1990–2014. (DOCX)
