# Supplementary material for: Disparities by race, age, and sex in the improvement of survival for lymphoma: Findings from a population-based study
Source: PLoS One. 2018 Jul 11;13(7):e0199745. doi: 10.1371/journal.pone.0199745 (PMC6040734; doi:10.1371/journal.pone.0199745)
Supplement: S1 Table — (DOCX) [file pone.0199745.s001.docx]

**S1 Table.** **Multivariate-Adjusted Hazard Ratios (HRs) and 95% CIs for Cancer-Specific Death (due to Lymphoma) by Stage and Age Group in 9 SEER Registries (1990-2009)**

|  | **Lymphoma** |  | **HL** |  | **NHL** |
| --- | --- | --- | --- | --- | --- |
| **Stage and age group, y** | **HR (95% CI)** |  | **HR (95% CI)** |  | **HR (95% CI)** |
| **Stage 1** |  |  |  |  |  |
| 20-49 | 0.569 (0.541-0.599) |  | 0.693 (0.572-0.840) |  | 0.541 (0.513-0.570) |
| 50-64 | 0.666 (0.635-0.699) |  | 0.804 (0.626-1.032) |  | 0.661 (0.622-0.702) |
| 65-74 | 0.719 (0.686-0.752) |  | 0.837 (0.679-1.031) |  | 0.714 (0.682-0.749) |
| 75-85 | 0.835 (0.811-0.859) |  | 1.053 (0.856-1.294) |  | 0.828 (0.794-0.864) |
| **Stage 2** |  |  |  |  |  |
| 20-49 | 0.695 (0.653-0.740) |  | 0.712 (0.635-0.797) |  | 0.654 (0.607-0.705) |
| 50-64 | 0.664 (0.627-0.703) |  | 0.655 (0.553-0.801) |  | 0.661 (0.622-0.705) |
| 65-74 | 0.752 (0.711-0.795) |  | 0.787 (0.632-0.981) |  | 0.750 (0.708-0.795) |
| 75-85 | 0.853 (0.811-0.896) |  | 0.906 (0.740-1.108) |  | 0.850 (0.807-0.895) |
| **Stage 3** |  |  |  |  |  |
| 20-49 | 0.779 (0.734-0.827) |  | 0.890 (0.782-1.013) |  | 0.700 (0.654-0.750) |
| 50-64 | 0.679 (0.646-0.713) |  | 0.665 (0.553-0.801) |  | 0.679 (0.645-0.715) |
| 65-74 | 0.741 (0.705-0.779) |  | 0.867 (0.729-1.032) |  | 0.730 (0.693-0.769) |
| 75-85 | 0.801 (0.764-0.839) |  | 0.824 (0.685-0.990) |  | 0.800 (0.762-0.839) |
| **Stage 4** |  |  |  |  |  |
| 20-49 | 0.702 (0.679-0.725) |  | 0.799 (0.722-0.883) |  | 0.694 (0.670-0.718) |
| 50-64 | 0.721 (0.700-0.742) |  | 0.795 (0.682-0.927) |  | 0.718 (0.697-0.740) |
| 65-74 | 0.773 (0.751-0.796) |  | 0.762 (0.644-0.901) |  | 0.774 (0.751-0.797) |
| 75-85 | 0.835 (0.811-0.859) |  | 0.918 (0.793-1.063) |  | 0.833 (0.809-0.857) |

**S2 Table. Baseline Demographic and Tumor Characteristics of Lymphoma Cancer Patients by Diagnosis Year in Nine SEER Registries, 1990-2014**

|  | **1990-1994** | | **1995-1999** | | **2000-2004** | | **2005-2009** | | **2010-2014** | |
| --- | --- | --- | --- | --- | --- | --- | --- | --- | --- | --- |
|  | N | % | N | % | N | % | N | % | N | % |
| **Age** |  |  |  |  |  |  |  |  |  |  |
| 20-49 | 6,141 | 19.71 | 6,250 | 20.06 | 6,261 | 20.09 | 6,505 | 20.88 | 6,003 | 19.27 |
| 50-64 | 3,849 | 13.46 | 4,475 | 15.65 | 5,533 | 19.35 | 6,849 | 23.96 | 7,883 | 27.57 |
| 65-74 | 3,883 | 17.51 | 4,163 | 18.77 | 4,042 | 18.22 | 4,546 | 20.50 | 5,547 | 25.01 |
| 75-85 | 3,444 | 16.23 | 4,086 | 19.26 | 4,509 | 21.25 | 4,652 | 21.92 | 4,528 | 21.34 |
|  |  |  |  |  |  |  |  |  |  |  |
| **Race** |  |  |  |  |  |  |  |  |  |  |
| White | 16,361 | 18.15 | 17,733 | 19.67 | 18,791 | 20.84 | 20,503 | 22.74 | 21,116 | 18.60 |
| African American | 1,466 | 15.67 | 1,673 | 17.88 | 1,913 | 20.45 | 2,291 | 24.49 | 2,496 | 21.52 |
| Asian or Pacific Islander | 977 | 13.46 | 1,205 | 16.60 | 1,518 | 20.91 | 1,788 | 24.63 | 2,219 | 24.40 |
| Other | 145 | 9.06 | 237 | 14.81 | 285 | 17.81 | 445 | 27.81 | 626 | 30.50 |
|  |  |  |  |  |  |  |  |  |  |  |
| **Sex** |  |  |  |  |  |  |  |  |  |  |
| Male | 10,549 | 17.02 | 11,356 | 18.32 | 11,992 | 19.34 | 13,467 | 21.72 | 14,632 | 23.60 |
| Female | 8,400 | 16.22 | 9,492 | 18.33 | 10,515 | 20.30 | 11,560 | 22.32 | 11,825 | 22.83 |
|  |  |  |  |  |  |  |  |  |  |  |
| **Marital status** |  |  |  |  |  |  |  |  |  |  |
| Single | 4,369 | 17.95 | 4,585 | 18.84 | 4,677 | 19.22 | 5,225 | 21.47 | 5,482 | 22.52 |
| Married | 9,679 | 16.48 | 10,839 | 18.45 | 11,772 | 20.04 | 12,790 | 21.78 | 13,655 | 23.25 |
| Other | 4,172 | 17.61 | 4,486 | 18.94 | 4,829 | 20.39 | 5,078 | 21.44 | 5,122 | 21.62 |
| Unknown | 729 | 10.37 | 938 | 13.35 | 1,229 | 17.49 | 1,934 | 27.52 | 2,198 | 31.27 |
|  |  |  |  |  |  |  |  |  |  |  |
| **Stage** |  |  |  |  |  |  |  |  |  |  |
| Stage I | 5,272 | 17.66 | 5,969 | 20.00 | 6,224 | 20.85 | 6,159 | 20.64 | 6,222 | 20.85 |
| Stage II | 2,954 | 14.73 | 3,540 | 17.65 | 4,141 | 20.65 | 4,669 | 23.28 | 4,749 | 23.68 |
| Stage III | 2,383 | 14.45 | 2,603 | 15.78 | 3,088 | 18.72 | 3,992 | 24.20 | 4,431 | 26.86 |
| Stage IV | 6,176 | 17.08 | 6,470 | 17.89 | 6,999 | 19.35 | 8,062 | 22.29 | 8,457 | 23.39 |
| N/A | 538 | 13.19 | 730 | 17.90 | 851 | 20.86 | 947 | 23.22 | 1,013 | 24.83 |
| Unknown | 1,626 | 22.74 | 1,536 | 21.49 | 1,204 | 16.84 | 1,198 | 16.76 | 1,585 | 22.17 |
|  |  |  |  |  |  |  |  |  |  |  |
| **Seer registry** |  |  |  |  |  |  |  |  |  |  |
| San Francisco-Oakland SMSA - 1973+ | 3,394 | 18.36 | 3,564 | 19.28 | 3,644 | 19.71 | 3,769 | 20.39 | 4,116 | 22.26 |
| Connecticut - 1973+ | 3,078 | 17.89 | 3,357 | 19.51 | 3,422 | 19.89 | 3,613 | 21.00 | 3,735 | 21.71 |
| Detroit (Metropolitan) - 1973+ | 3,173 | 18.06 | 3,321 | 18.90 | 3,607 | 20.53 | 3,707 | 21.10 | 3,764 | 21.42 |
| Hawaii - 1973+ | 736 | 15.60 | 821 | 17.40 | 879 | 18.63 | 1,072 | 22.72 | 1,211 | 25.66 |
| Iowa - 1973+ | 2,497 | 17.67 | 2,609 | 18.46 | 2,753 | 19.48 | 3,070 | 21.72 | 3,206 | 22.68 |
| New Mexico - 1973+ | 907 | 14.37 | 1,095 | 17.35 | 1,225 | 19.41 | 1,495 | 23.69 | 1,590 | 25.19 |
| Seattle (Puget Sound) - 1974+ | 2,694 | 15.08 | 3,074 | 17.21 | 3,527 | 19.75 | 4,191 | 23.47 | 4,374 | 24.49 |
| Utah - 1973+ | 1,037 | 13.81 | 1,270 | 16.91 | 1,459 | 19.43 | 1,825 | 24.30 | 1,919 | 25.55 |
| Atlanta (Metropolitan) - 1975+ | 1,433 | 14.35 | 1,737 | 17.39 | 1,991 | 19.93 | 2,285 | 22.88 | 2,542 | 25.45 |
